# Fabrication of Waterproof Artificial Compound Eyes with Variable Field of View Based on the Bioinspiration from Natural Hierarchical Micro–Nanostructures

**DOI:** 10.1007/s40820-020-00499-x

**Published:** 2020-08-15

**Authors:** Peilin Zhou, Haibo Yu, Ya Zhong, Wuhao Zou, Zhidong Wang, Lianqing Liu

**Affiliations:** 1 State Key Laboratory of Robotics, Shenyang Institute of Automation, Chinese Academy of Sciences, Shenyang, 110016 People’s Republic of China; 2 Institutes for Robotics and Intelligent Manufacturing, Chinese Academy of Sciences, Shenyang, 110169 People’s Republic of China; 3 University of Chinese Academy of Sciences, Beijing, 100049 People’s Republic of China; 4 Department of Advanced Robotics, Chiba Institute of Technology, Chiba, 275-0016 Japan

**Keywords:** Bioinspired, Hierarchical MLAs and NLAs, Waterproof artificial compound eyes, E-jet printing, Microfluidics chip

## Abstract

Planar and curved microlens arrays (MLAs) are the key components of miniaturized microoptical systems. In order to meet the requirements for advanced and multipurpose applications in microoptical field, a simple manufacturing method is urgently required for fabricating MLAs with unique properties, such as waterproofness and variable field-of-view (FOV) imaging. Such properties are beneficial for the production of advanced artificial compound eyes for the significant applications in complex microcavity environments with high humidity, for instance, miniature medical endoscopy. However, the simple and effective fabrication of advanced artificial compound eyes still presents significant challenges. In this paper, bioinspired by the natural superhydrophobic surface of lotus leaf, we propose a novel method for the fabrication of waterproof artificial compound eyes. Electrohydrodynamic jet printing was used to fabricate hierarchical MLAs and nanolens arrays (NLAs) on polydimethylsiloxane film. The flexible film of MLAs hybridized with NLAs exhibited excellent superhydrophobic property with a water contact angle of 158°. The MLAs film was deformed using a microfluidics chip to create artificial compound eyes with variable FOV, which ranged from 0° to 160°.

**Electronic supplementary material:**

The online version of this article (10.1007/s40820-020-00499-x) contains supplementary material, which is available to authorized users.

## Highlights

- Bioinspired by natural superhydrophobic surface with hierarchical micro–nanostructures, waterproof artificial compound eyes with variable field of view (FOV) were fabricated by a simple and effective manufacturing method.
- The fabricated artificial compound eyes exhibited excellent waterproof property, tunability of the FOV and optical performance.

**Electronic supplementary material:**

The online version of this article (10.1007/s40820-020-00499-x) contains supplementary material, which is available to authorized users.

## Graphical abstract

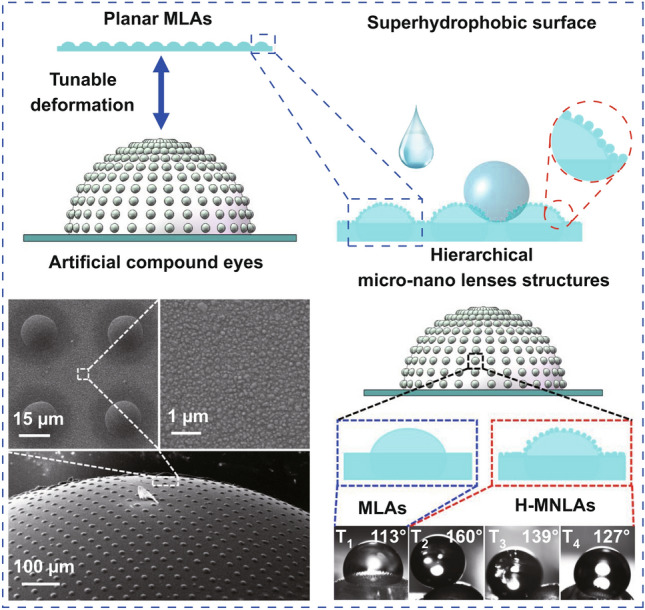

## Introduction

In recent years, the development of microlens arrays (MLAs), including planar and curved MLAs, has drawn significant attention owing to the wide range of applications of MLAs in interdisciplinary and microoptical fields [1–3]. The compound eyes of insects (e.g., dragonfly, honeybee, and moth) are composed of numerous microeyes and possess several unique properties such as a wide field of view (FOV) and high-sensitivity detection. Taking bioinspiration from insect eyes, recent researches have explored the use of curved MLAs as artificial compound eyes in diverse applications, such as fast motion tracking [4], digital cameras [5], medical endoscopy imaging [6], and wide field imaging [7].

Various methods have been investigated for the fabrication of planar and curved MLAs. Many conventional methods are applied to manufacturing planar MLAs such as laser direct writing [8], laser-induced writing [9, 10], polymer swelling [11], transfer imprinting [12], and inkjet printing [13]. In contrast, a three-dimensional (3D) processing capacity is required in the manufacturing methods to fabricate curved MLAs as artificial compound eyes. In this context, laser direct writing is suitable for fabricating curved MLAs directly with high precision [14]. However, the high cost and time required for the complicated 3D fabrication process limit its fabrication efficiency for widespread applications. On the other hand, based on the planar MLAs manufactured via conventional methods, artificial compound eyes can be fabricated indirectly by deforming planar MLAs to curved MLAs using vacuum absorption and thermoplastic molding [15–17]. The deformation of MLAs film is a simple and efficient method for fabricating artificial compound eyes. However, there is little scope to adjust the physical parameters of the eyes, because the flexibility to deform the shape and parameters of the curved MLAs are limited by the fixed profile of the deformation molds.

In order to satisfy versatile applications, high integration, miniaturization, and adjustability have proved desirable qualities for the development of microoptical systems [18]. Furthermore, waterproofness has become an urgent requirement for the application of advanced microoptical systems in humid environments, such as advanced tissue endoscopes [19]. Bioinspired by the hierarchical micro–nanostructures, which resulting increate the superhydrophobic surface (the water contact angle (WCA) of which > 150°) of lotus leaf [20], the fabrication of MLAs consisting of hierarchical micro–nanostructures with the optical property may be a simple and effective approach for realizing waterproof microoptical systems. Wang et al. have realized the fabrication of artificial compound eyes via the deformation of MLAs with hierarchical micro–nanostructures [15, 16]. Meanwhile, tunable optical systems have been investigated using different methods in several fields with unique potential applications [21], e.g., a lens with a tunable focal length has been developed using a microfluidics chip for optofluidic research [22, 23]. At the same time, Sun et al. have carried out an advanced study of smart compound eyes [24], demonstrating that artificial compound eyes with tunable parameters are necessary for practical applications. For example, a tunable curved surface with a variable FOV assists zoom variation for various imaging requirements. However, at present, due to the complexity of the manufacturing process, the simple and effective fabrication of waterproof artificial compound eyes with tunable parameters faces significant challenges. An effective method of realizing planar MLAs with superhydrophobic structures and a tunable approach to realize the flexible deformation of planar MLAs to create curved MLAs, are both issues that must be solved to realize the simple fabrication of waterproof artificial compound eyes with a variable FOV.

As an ultra-high-resolution printing technique, electrohydrodynamic jet (E-jet) printing has been demonstrated to be a reliable method for maskless and direct manufacturing on various substrates at a large scale [25]. Due to the flexibility of E-jet printing and its ability to fabricate structures with feature ranging from microscale to nanoscale sizes, its use has been developed for various applications, such as nanomaterials patterning [26], flexible electronic devices [27], microfluidic/optical devices [28], and bio-sensors [29]. Based on our previous study of the cross-scale fabrication of micro-/nanolens arrays [30], E-jet printing will provide an effective tool for fabricating desired MLAs and NLAs on a tunable substrate.

In this paper, we present a novel method for the fabrication of waterproof artificial compound eyes with a variable FOV, which are inspired by the natural superhydrophobic structures of a lotus leaf and assisted by a microfluidics chip. Based on the investigation of numerical simulation and theoretical analysis of the electrical field for the E-jet printing, the optimized hierarchical MLAs integrated with NLAs were fabricated on a polydimethylsiloxane (PDMS) film substrate by the E-jet printing of an ultraviolet (UV) curable adhesive. The hydrophobicity of the MLAs film was improved to realize the waterproof property by controlling the size and density of the NLAs. Meanwhile, the flexible MLAs film was deformed from a planar surface to a curved surface via the integrated microfluidics chip. The tunable mechanism enabling the FOV of the artificial compound eyes to be adjusted was analyzed by controlling the volume of the liquid injected via the microfluidics chip. Finally, the waterproofness, tunability of the FOV, and optical performance of the artificial compound eyes were investigated experimentally. The results demonstrate that the simple and effective approach to fabricate artificial compound eyes presented herein has the potential to be utilized in diverse microoptical applications.

## Experimental Section

### Experimental Apparatus

The E-jet printing system consisted of four parts: a motion stage, a power supply, an observation system, and a printing nozzle. A computer-controlled three-axis motorized translation stage (Newport, USA) with a conductive metal substrate acted as the motion stage. This was used to control the programmed movement, with route planning based on LabView, for drop-on-demand (DOD) and continuous route (CR) printing patterns during the fabrication process. A high-voltage generator (Dongwen, China) acted as the power supply, and it was connected between the printing nozzle and conductive substrate to provide a high electric field for the printing process. An optical microscope (Navitar, USA) equipped with a charge-coupled device (CCD) camera (Point Gray, Canada) acted as the observation system, which was used to monitor the gap distance and ejection state during the printing process. During the experiment, sharp glass microcapillaries with inner diameters of 1, 2, 10, 40, and 60 μm were manufactured as printing nozzles via a pipette puller (P-97, Sutter, Inc., USA).

### Fabrication Process of the Tunable Artificial Compound Eyes

There were three steps in the fabrication process: (1) fabrication of the PDMS film; (2) fabrication of hierarchical MLAs and NLAs on the PDMS film; (3) tunable deformation of the MLAs film via the integrated microfluidics chip.

For the fabrication of the PDMS film, a liquid PDMS elastomer (Sylgard 184, Dow Corning, USA) and a curing agent were mixed with a weight ratio of 10:1. Then, the PDMS was spin-coated onto a glass substrate (150 μm thick) surrounded by a four-sided fenced structure with a height of 50 μm at a speed of 1000 rpm for 30 s. Subsequently, the PDMS film was cured at 100 °C for 30 min. The average thickness of the film was 50 μm, and it was used as the soft substrate.

During the experiment, the UV-curable adhesive (NOA61, Thorlabs, Inc.) was used as the printing material for fabricating MLAs and NLAs. The basic parameters of the UV-curable adhesive are density of 1.02 g cm^−3^, refractive index of 1.56, and viscosity of 300 cps. After printing on the PDMS film substrate, micro-/nanodroplet arrays were precured via 10 s of exposure under 355 nm UV light at 1000 mJ cm^−2^. The droplets were completely solidified after 30 min of UV exposure.

The microfluidics chip was fabricated using PDMS structures, which were created by the replication of polymethylmethacrylate micromolds. These molds were fabricated using a computer numeric controlled (CNC) engraving machine (EGX-400, Roland Inc., Japan). Subsequently, the tunable deformation process for the MLAs film was realized via the following steps: First, the MLA film was peeled off from the glass slide substrate. Second, the MLAs film was assembled at the hole at the center channel of a microfluidics chip and then encapsulated to be integrated with the chip. Third, a liquid (NOA61 UV-curable adhesive) was injected into the channel of the microfluidics chip, and the fluxion and volume of the liquid were adjusted precisely via two syringe pumps (Longer, China). Finally, the soft MLAs film was deformed from a planar surface to a curved surface to create the artificial compound eyes. The radius of curvature and FOV of the eyes were adjusted by changing the volume of the injected liquid.

### Characterization

The geometric size and surface morphologies of the fabricated planar and curved MLAs were characterized and analyzed using a commercial atomic force microscopy (AFM) system (Dimension Icon, Bruker Nano, Inc., USA) and a scanning electron microscope (SEM) (Quanta 400 FEG, FEI, USA). The optical micrographs for the characterization of the optical performance of the planar and curved MLAs were obtained by utilizing an optical microscope (HIROX KH-7700, Japan) equipped with a CCD camera (Point Gray, Canada). The WCAs of the fabricated MLAs film were characterized using a homebuilt measurement system. The system contained the following four major components: a three-axis motion stage (Newport, USA) that acted as a sample stage, a syringe pump (Longer, China) with a microinjector that acted as a liquid titration system, a horizontal-view optical microscope (Navitar, USA) equipped with a CCD camera (Point Gray, Canada), and a light source (Fig. S1). Five measurement results were used to obtain the average WCAs for different samples.

## Results and Discussion

### Design of Bioinspired Compound Eyes with Waterproof Property

**Fig. 1:**
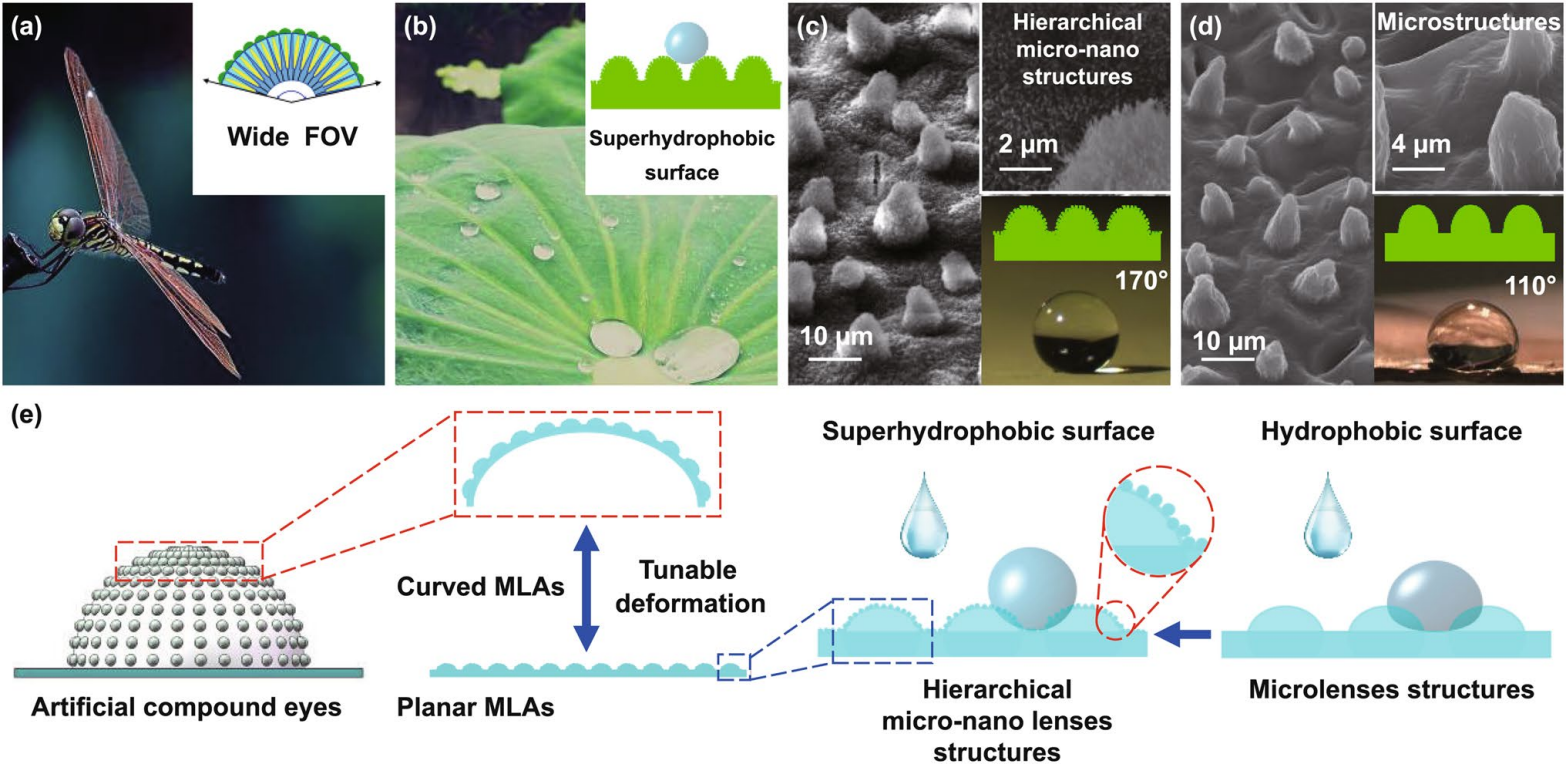
Illustration of artificial compound eyes inspired by the compound eyes of dragonfly and the superhydrophobic surface of lotus leaf. **a** A dragonfly, whose eyes have a wide FOV (see inset). **b** A lotus leaf, whose surface morphology leads to its superhydrophobic property. **c** SEM images of the lotus leaf with nanotomenta. **d** SEM images of the lotus leaf without nanotomenta. **e** Schematic diagram for the fabrication of the waterproof artificial compound eyes with variable FOV

Inspired by the compound eyes of dragonfly and the superhydrophobic property reported for lotus leaf, we first investigated the morphology and corresponding wetting behavior of the compound eyes of dragonfly and lotus leaf. Figure 1a shows a dragonfly, the compound eyes of which are multifaceted, consisting of thousands of ‘microeyes’ (known as ommatidia) that form a dome-like surface, and have outstanding imaging capabilities. However, as shown in (Fig. S2), the WCA measured on the surface of dragonfly eyes is approximately 80°. Therefore, the surface wetting behavior of the eyes proves that the biological prototype of which is unsuitable for fabricating waterproof artificial compound eyes. The 45° tilt view and cross-sectional view used for the SEM images of the compound eyes show numerous homogeneously distributed ommatidia (Fig. S3), each with an average diameter of approximately 40 μm. Figure 1b shows a lotus leaf, with SEM images (at 45° tilt view) of the leaf shown in Fig. 1c. There are numerous microscale papillae, covered with nanoscale tomenta, randomly distributed on the surface of the leaf. The diameter of the microscale structures comprising the micropapillae is in the range of approximately 5–20 μm, while the diameters of the nanoscale structures comprising the nanotomenta is in range of 100–200 nm (shown in the inset of Fig. 1c). Significantly, the WCA measured on the surface of the lotus leaf is approximately 170°. This shows that the leaf has excellent superhydrophobicity, and it is a suitable biological prototype for creating a waterproof surface. Even though hierarchical micro–nanostructures are the key factors in creating the superhydrophobicity of the lotus leaf, the individual effects of the microstructures and nanostructures have not been investigated. To examine their effect, the nanotomenta of a lotus leaf were removed via heat treatment at 120 °C for 30 min after the conventional drying process, as shown in the schematic and images (Fig. S4). Figure 1d shows the corresponding SEM image in 45° tilt view of the heat-treated surface. With only micropapillae remaining on the surface of the leaf after the removal of the tomenta, the WCA of the leaf decreased to 110° (shown in the inset of Fig. 1d). Therefore, we can conclude that the nanostructures enhance the hydrophobicity of the lotus leaf. As a consequence, the combined properties of the compound eyes of dragonfly and the superhydrophobic lotus leaf led to their selection as the biological prototypes for developing waterproof artificial compound eyes with a variable FOV. This included the fabrication of a waterproof flexible MLAs film that subsequently underwent a tunable deformation, as shown in the schematic in Fig. 1e. The soft PDMS film and the hierarchical MLAs and NLAs structures covering it constituted the complete MLAs film, the hydrophobicity of which was improved by the hybridized NLAs.

### Fabrication Process of the Artificial Compound Eyes

**Fig. 2:**
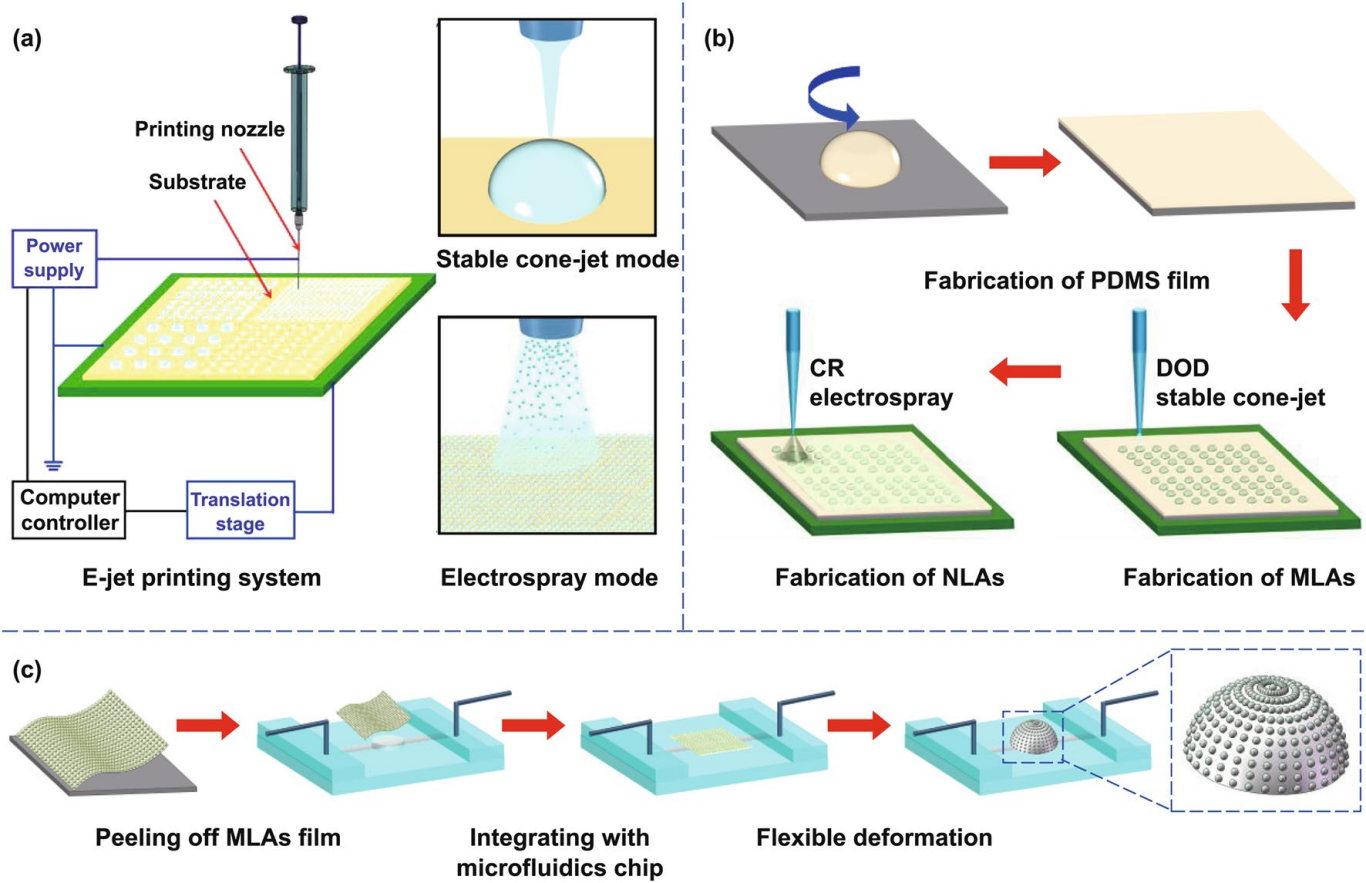
Schematic illustration of the fabrication process for the waterproof artificial compound eyes with variable FOV. **a** Fabrication of hierarchical micro-/nanodroplets via the stable cone-jet and electrospray printing modes using an E-jet printing system. **b** Fabrication of hierarchical MLAs and NLAs on the flexible PDMS film. **c** Flexible deformation of the MLAs film using an integrated microfluidics chip to fabricate artificial compound eyes

The manufacturing equipment and fabrication process for the artificial compound eyes are illustrated in Fig. 2. The E-jet printing system with the stable cone-jet and electrospray printing modes is shown in Fig. 2a. During the printing process, the applied voltage provides an extremely high electric field between the tip and ground electrode. Electrostatic force pushes the ink to overcome the multiple coupling forces, and the ink is ejected as micro-/nanodroplets from the apex of the nozzle. Furthermore, according to electrohydrodynamic (EHD) theory, the ejection mode of droplets varies with the electric field. Thus, ink can be ejected in various states owing to the change in the printing mode with the increase in the electric field. These states include the dripping state, microdripping state, stable cone-jet state, unstable cone-jet state, multijet state, and electrospray state [31]. The stable cone-jet mode and electrospray mode were selected to print the NOA61 UV-curable adhesive for the fabrication of MLAs and NLAs, respectively, because of the stability of the stable cone-jet mode and the high efficiency of the electrospray mode.

The entire process consisted of two steps, i.e., the fabrication of the MLAs film, which consisted of the hierarchical MLAs and NLAs on the PDMS film, and the deformation of the MLAs film. As shown in Fig. 2b, PDMS was dropped and spin-coated on a glass slide substrate and then cured to prepare the PDMS film. Next, MLAs were DOD printed onto the surface of the PDMS film via the stable cone-jet mode of E-jet printing system (Fig. S5), and cured via UV light for 30 min. Thereafter, NLAs were CR printed on the surface of the MLAs film via the electrospray printing mode and cured via UV light. Subsequently, the flexible MLAs film was deformed to create artificial compound eyes with a variable FOV according to the process shown in Fig. 2c (Fig. S6).

**Fig. 3:**
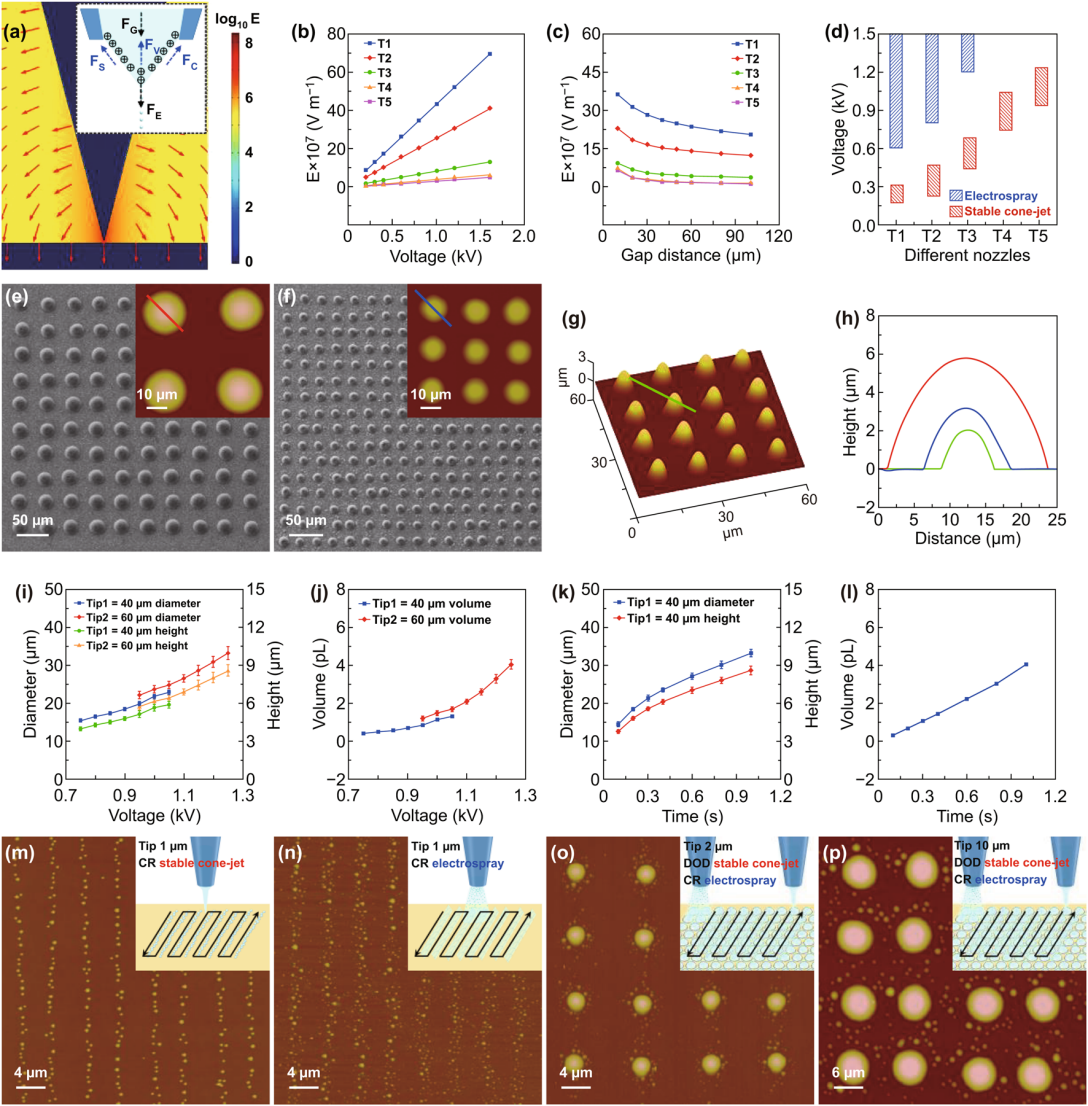
Hierarchical fabrication of MLAs and NLAs by E-jet printing. **a** Finite-element method model of the electric field (E) distribution around the nozzle tip and substrate during the ejection process. **b** and **c** The variation in E at the apex of the printing nozzles, with different inner diameters, for various voltages and gap distances, respectively. **d** E-jet printing modes for NOA61 using different printing nozzles and voltages. **e–h** SEM and AFM images of MLAs with different sizes obtained via the stable cone-jet mode. **i–l** Diameter, height, and volume of the microlenses as functions of the amplitude and duration of applied voltage. **m** and **n** AFM images of NLAs fabricated via the stable cone-jet and electrospray modes, respectively. **o** and **p** AFM images of various hierarchical MLAs combined with NLAs

For the numerical and experimental analyses of the ejection modes for the printing of hierarchical MLAs and NLAs, a simulation model for the spatial distribution and intensity of the electric field between the printing nozzle tip and substrate was developed using COMSOL Multiphysics commercial software. The angle and inner diameter of the tip were set as 30° and 1 μm, respectively. The gap distance between the tip and substrate was maintained at 40 μm, and the applied voltage was 0.5 kV. Figure 3a shows the magnitude and direction vectors (white arrows) of the electric field (E) between the tip and substrate. According to EHD theory [32], the inset schematic illustrates that the droplets are ejected through the multiple coupling of the electric field force (*F*_E_), surface tension (*F*_S_), capillary force (*F*_C_), viscous force (*F*_V_), and gravity force (*F*_G_). The detailed statistics for variation of E at the apex of the tip with different parameters are shown in Fig. 3b, c, where T1, T2, T3, T4, and T5 denote the printing nozzles with inner tip diameters of 1, 2, 10, 40, and 60 μm, respectively. Although E increases with increasing voltage, it decreases as the inner tip diameter and gap distance increase. As shown by the statistics in Fig. 3d, when the gap distance was 40 μm and the NOA61 was used as the ink, the printing modes for various printing nozzles were controlled using different voltage ranges. The voltages required for the stable cone-jet mode for the printing nozzles with diameters of 1, 2, 10, 40, and 60 μm, and the electrospray mode for the nozzles with diameters of 1, 2, and 10 μm were investigated, respectively. The required voltage increases with the diameter of the nozzle for the same printing mode. Moreover, the electrospray mode requires a higher voltage compared to the stable cone-jet mode for an equivalent nozzle diameter.

As the SEM and AFM images shown in Fig. 3e–g, the average diameters of the microlenses are 22.64 ± 0.88, 12.16 ± 0.43, and 7.74 ± 0.29 μm, respectively. The homogeneous MLAs with different sizes were DOD printed via the stable cone-jet mode (Video S1), based on the corresponding scanned profiles shown in Fig. 3h; the diameter/height (*d*/*h*) ratios of the different lenses were constant at ~ 3.85 due to the wettability of the substrate being consistent. The focal length and numerical aperture (NA) are the key optical parameters of a microlens, and they are determined by the morphological parameters (diameter and height) of the microlens. Hence, the parameters for the adjustment of diameter and height were explored. As the d/h ratio is constant, the volume of the microlens can be calculated as a more intuitive parameter. The relationships between the diameter, height, and volume of the microlens, and the amplitude and duration of the applied voltage were investigated, respectively. As shown in Fig. 3i, j, when the duration of the applied voltage is 1 s, the diameter, height, and volume of the microlens increase with the amplitude of the applied voltage. For an equivalent applied voltage, the microlens fabricated by the nozzle with inner diameter of 60 μm is larger than that fabricated by the nozzle with inner diameter of 40 μm. Figure 3k, l shows that the diameter, height, and volume of the microlens increased with the duration of the applied voltage when the inner diameter of the nozzle and the amplitude of the applied voltage were 40 μm and 0.9 kV, respectively.

Even though we have realized the DOD fabrication of NLAs via the stable cone-jet mode in our previous work [30], the size and position distribution of each nanolens could be controlled precisely using optimized parameters (Fig. S7). Figure 3m shows that the NLAs contain ~ 500 nanolenses, which were CR fabricated in ~ 0.2 s using the nozzle with an inner diameter of 1 μm via the stable cone-jet mode (see inset schematic). The CR fabrication is not as uniform as the DOD fabrication, but its manufacturing efficiency is higher. Based on previous researches, both the uniform and random distribution of microstructures and nanostructures can enhance the hydrophobic performance of a surface [33, 34]; especially, in the basic of microstructures, hierarchical micro–nanostructures could remarkably enhance the hydrophobic performance [35, 36]. Therefore, the superhydrophobicity of the substrate surface is determined by the sizes and densities of nanostructures rather than the uniformity of their distribution. By contrast, the CR fabrication of NLAs via the electrospray mode was more efficient (Video S2), as shown in Figs. 3n and S8; NLAs with more than 5,000 and 10,000 randomly distributed nanolenses were fabricated in ~ 0.1 and ~ 0.2 s, respectively. After investigating the separate fabrication of MLAs and NLAs, we have demonstrated the hierarchical fabrication of MLAs and NLAs by utilizing E-jet printing nozzles with the same tip diameters (2 and 10 μm) via different printing modes and parameters, as shown in Fig. 3o, p; the homogeneous MLAs were distributed regularly, while NLAs were distributed randomly. Due to the high resolution as a distinct advantage of E-jet printing, the diameter of a printed droplet is smaller than the diameter of the printing nozzle. Thus, MLAs with a diameter of more than 10 μm cannot be fabricated rapidly by utilizing the 10-μm diameter nozzle unless printing time is increased. Meanwhile, although the larger printing nozzles (with inner diameter larger than 10 μm) are capable of droplet ejection at the nanoscale, increasing the applied voltage results in more anabatic phenomena including droplet fusion and the Taylor cone shake effect, which are unsuitable for stable the fabrication of NLAs. To improve the manufacturing efficiency and outcome, the nozzles with diameters of 40 and 60 μm were used to fabricate MLAs, while the nozzles with diameters of 1, 2, and 10 μm were used to fabricate NLAs.

### Characterization of the Waterproof Ability of the Artificial Compound Eyes

**Fig. 4:**
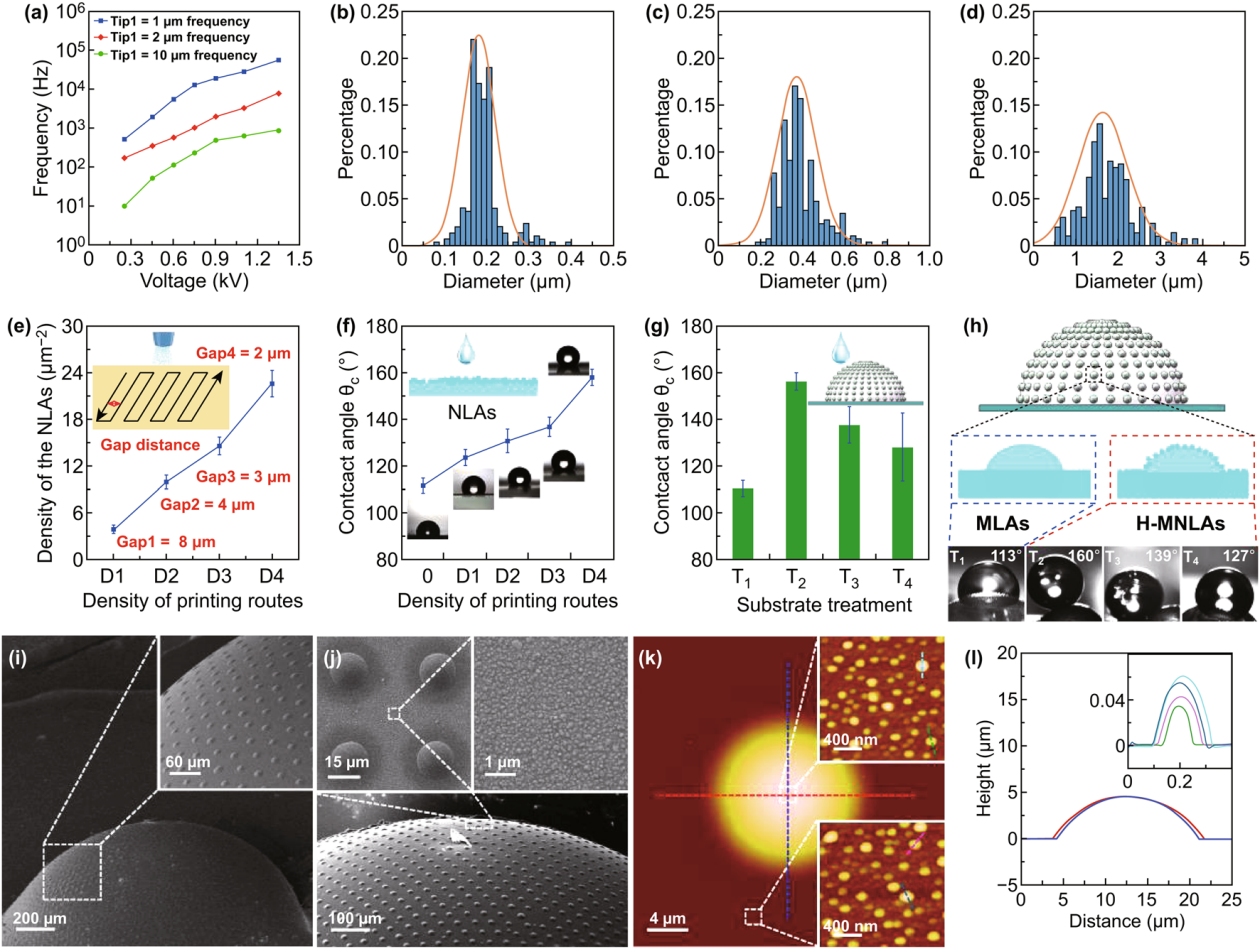
Characterization and statistical analysis of the fabricated artificial compound eyes. **a–d** Statistical analysis of the fabrication frequency and diameter distribution of the nanolenses printed by nozzles with diameters of 1, 2, and 10 μm. **e** Relative densities of nanolenses as a function of the densities of printing routes for a nozzle with inner diameter of 1 μm. **f** WCAs of the surface of PDMS films covered with different densities of nanolenses printed via a nozzle with inner diameter of 1 μm. The inset images show the WCA measurements for the corresponding PDMS film. **g** Statistics for the WCAs of the artificial compound eyes fabricated using a flexible MLAs film. The MLAs film was modified through different treatments (T1–T4) via nanolenses fabricated with different parameters. **h** The corresponding images of the WCA measurements for the surface of artificial compound eyes fabricated using T1–T4, respectively. **i** and **j** SEM images of the artificial compound eyes fabricated utilizing a flexible MLAs film. The inset images in (**i**) and (**j**) show the magnified details of the hierarchical micro–nanolenses. **k** and **l** Two-dimensional AFM image and the corresponding AFM profiles of the hierarchical micro–nanolenses. The inset images in (**l**) are the corresponding profiles of the nanolenses shown in (**k**)

Following our research to optimize their design and fabrication, we characterized the wettability of the fabricated artificial compound eyes. Nanolenses were fabricated using the following parameters: a gap distance of 40 μm, with voltages of 0.3–1.25 kV applied to the nozzles with diameters of 1, 2, and 10 μm. Figure 4a shows that the fabrication frequency of nanolenses increases with voltage but decreases as the inner diameter of the nozzle increases. Additionally, the volume of nanolenses decreases as voltage increases but increases with the inner diameter of the nozzle (Fig. S9). Figure 4b–d shows the diameter distributions of the nanolenses when a voltage of 1.25 kV is applied to the nozzles; NLAs were fabricated via CR printing under the electrospray printing mode, and the corresponding SEM and AFM images are shown in (Fig. S10). It can be concluded that the diameter and the standard deviation of the sizes of the nanolenses increase with the inner diameter of the printing nozzle. This phenomenon is due to the fusion of ejected nanodroplets caused by the Ostwald ripening effect [37]. In addition, the nonnegligible charge in a heterogeneous electric field affects nanodroplets [38], with the fusion of nanodroplets contributing to the decrease in the size deviation of the nanolenses, and the fusion phenomenon of nanodroplets is exacerbated in a higher electric field. As a result, the standard deviation of the sizes of nanolenses increases with the inner diameter of the printing nozzle, which results in the decrease in electrical field. The density of NLAs can be adjusted by controlling the densities (D) of printing routes, which was controlled by adjusting the gap distance between adjacent routes. The CR fabrication of NLAs with different densities via the electrospray mode using the nozzle with a diameter of 1 μm was investigated in detail. As shown in Fig. 4e, the density of NLAs increases with the densities of the printing routes. Here, the gap distances of D1, D2, D3, and D4 were 8, 4, 3, and 2 μm, respectively. The average diameter of the nanolenses forming the arrays ranges from 100 to 300 nm, and the densities corresponding to the aforementioned-gap distances were 3.9, 9.9, 14.4, and 22.6/μm^2^, respectively.

The characterization of the wettability of the PDMS film modified with NLAs fabricated using the nozzle with an inner diameter of 1 μm is shown in Fig. 4f. According to the analyses of previous research [15, 36], denser nanolenses increase the surface roughness of the PDMS film, which results in the enhanced hydrophobic performance of the surface. The WCAs of the surface of the PDMS films increase with increasing densities of nanolenses (determined by the distances D1–D4). The inset images for Fig. 4f clearly show the variation in the WCAs of the PDMS films with different densities of nanolenses. Meanwhile, the wettability of the artificial compound eyes fabricated by utilizing the flexible MLAs film with various modifications of NLAs was investigated. The MLAs were fabricated by employing the nozzle with an inner diameter of 40 μm. T1 indicates the MLAs film without NLAs, and T2, T3, and T4 indicate the MLAs film with the densest NLAs distributions fabricated using the nozzles with inner diameters of 1, 2, and 10 μm, respectively. As the statistics and corresponding images shown in Fig. 4g and 4h, we can conclude that the artificial compound eyes fabricated using the MLAs film with hierarchical MLAs and NLAs (H-MNLAs) exhibit better hydrophobicity than the MLAs film fabricated using only MLAs. This proves that artificial compound eyes fabricated using our method have strong potential for use in applications that require wide FOV imaging in humid or even aqueous environments. The average WCA of the artificial compound eyes increases from 113° to 158° via the modification of NLAs fabricated by nozzles with inner diameter of 1 μm. Besides, the enhancement of hydrophobicity via NLAs decreases as the inner diameter of used printing nozzle increases. This phenomenon is due to the stability of fabrication of nanolenses decreasing as the inner diameter of the printing nozzle increases; this results in the larger size, larger deviation of size, and lower relative density of NLAs (Fig. S9), which are incompatible with increasing the hydrophobicity. Figure 4i shows the SEM image of the artificial compound eyes fabricated by utilizing the deformation method for MLAs film described herein. The height, diameter, radius of curvature, and FOV of the eyes are 0.68 mm, 1.6 mm, 0.81 mm, and 161.5°, respectively. The inset high-resolution SEM images in Fig. 4i, j reveal further that the hierarchical micro–nanolenses are distributed uniformly and closely on the spherical surface, which is beneficial for various practical and potential applications. The hierarchical micro–nanolenses were fabricated using the nozzles with inner diameters of 40 and 1 μm, respectively, with the corresponding AFM images and the scanned profiles shown in Fig. 4k, l (nanolenses shown in the insets). The average diameters and heights are 17.55 and 4.63 μm for the microlenses, and 183.8 and 48.3 nm for the nanolenses.

### Deformation Mechanism and Simulation of Focusing Properties

**Fig. 5:**
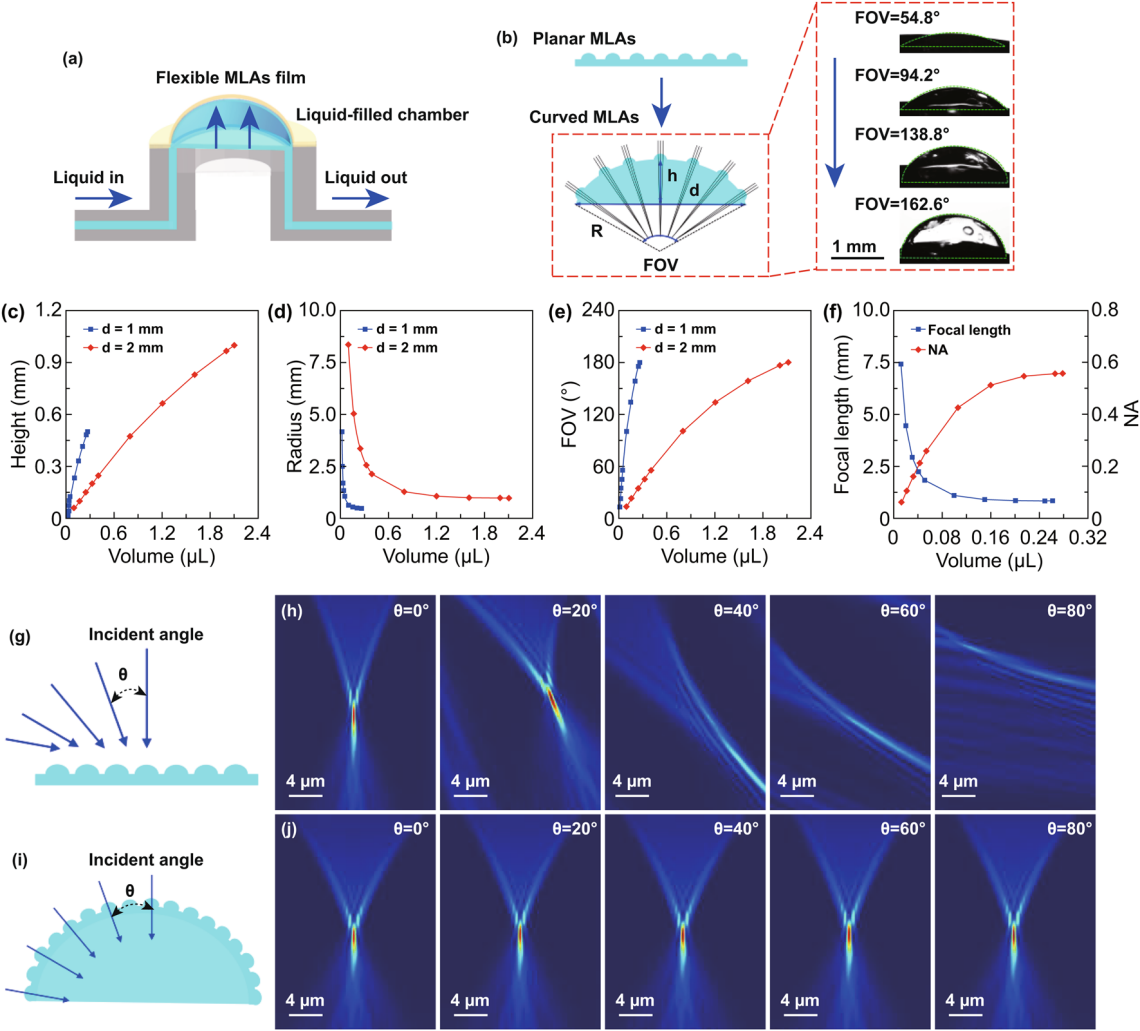
Schematic and analysis of the deformation mechanism and focusing properties of the artificial compound eyes. **a** Deformation mechanism of the artificial compound eyes via a microfluidics chip. **b** Deformation of planar MLAs to curved MLAs. **c** and **d** Height and the radius of curvature of the tunable artificial eyeball as functions of the volume of liquid injected in the chamber, respectively. **e** and **f** FOV, focal length and NA of the eyeball as functions of the volume of injected liquid, when the diameter of eyeball is 1 mm. **g–j** FDTD simulation results for the light intensity distribution of focal spots via single microlens of planar MLAs, (**g**) and (**h**), and curved MLAs, (**i**) and (**j**), at various incident angles

Figure 5a illustrates the deformation mechanism of the tunable artificial compound eyes, which consist of the flexible MLAs film and a liquid-filled chamber between the film and the hole at the center channel of the microfluidics chip. The liquid-filled chamber acted as the tunable component of the artificial compound eyes. When the chamber was filled with the injected liquid (NOA61), as illustrated in Fig. 5b, the MLAs film was extruded and deformed from a planar surface to a curved surface. The diameter of the eyeball depends on the diameter of the hole. The height and radius of curvature of the eyeball were adjusted by varying the volume of the injected liquid. According to optical theory and geometry, the volume of the chamber/eyeball (*V*), radius of curvature (*R*), FOV, focal length (*f*), and NA of the eyeball can be determined using the following formula:

1 $$ V \, = \, \frac{{\pi h\left( {3d^{2} + \, 4h^{2} } \right)}}{24} $$

2 $$ R \, = \, \frac{{ \, d^{2} + \, 4h^{2} }}{8h} $$

3 $$ {\text{FOV = 2arcsin}}\left( {\frac{4dh}{{d^{2} + \, 4h^{2} }}} \right) $$

4 $$ f \, = \, \frac{{h^{2} + \, d^{2} /4}}{{2h\left( {n - 1} \right)}} $$

5 $$ {\text{NA = }}d/(2f) $$

where *h* and *d* are the height and diameter of the eyeball, respectively, and *n* is the refractive index of the liquid. As the height and radius of curvature are altered by changing the volume (*V*) of the liquid in the chamber, this results in a tunable FOV. Figure 5b shows the cross-sectional optical images of four different configurations of the tunable artificial compound eyes with a diameter of 2 mm, and corresponding FOVs of 54.8°, 94.2°, 138.8°, and 162.6° (Fig. S11 and Video S3).

According to theoretical deduction and numerical calculation, the FOV is determined by the height and radius of curvature of the eyeball, which depend on the volume and diameter of the eyeball. As the diameter of the eyeball is constant, its height can be adjusted based on the tunable volume. Similarly, the radius of curvature and FOV of the eyeball can be tuned precisely by controlling the tunable volume. Furthermore, the eyeball can function as a tunable lens (Fig. S12), with precise tunability of the focal length and NA also realized via the tunable volume. According to the above analysis, and supported by the plots shown in Fig. 5c–f, the height, FOV, and NA increase with increasing volume, while the radius of curvature and focal length decrease as volume increases. When the diameter was set as 1 mm and the volume of chamber was increased from 0.012 to 0.26 μL, the height of the eyeball increased from 0.03 to 0.5 mm, the corresponding radius of curvature decreased from 4.18 to 0.5 mm, the FOV increased from 13.73° to 180°, the focal length decreased from 7.47 to 0.89 mm, and the NA increased from 0.07 to 0.56.

Meanwhile, theoretical models were established to simulate the focused light intensity distribution of 3D models via the finite-difference time-domain (FDTD) method. The simulations were performed using the following parameters: The light source was defined as a parallel wave (*λ* = 500 nm, polarization angle of 0°), and 15 μm away from the center of sphere, with perfectly matched layer boundary conditions of grid size (mesh accuracy: second level, minimum mesh step: 0.00025 μm). As shown in Fig. 5g, i, parallel white light was selected as the incident beam at various angles (0°, 20°, 40°, 60°, and 80°) to simulate the intensity distribution for a single microlens of the planar and curved MLAs (theoretical FOV was 160°), respectively. The refractive indices of NOA61 (microlens and eyeball), PDMS, and air are *n*_1_ = 1.56, *n*_2_ = 1.41, and *n*_3_ = 1, respectively. The basic height and diameter of a single microlens were set as 5 and 20 μm, respectively. Figure 5h, j compares the theoretical FDTD simulation results of the light intensity distribution of focal spots at various incident angles. The light intensity distribution results corresponding to the planar MLAs show evident distortion as the incident light angle is tilted away from normal incidence, with the distortion increasing with the incident angle. In contrast, there is no distortion in the light intensity distribution corresponding to the curved MLAs in response to increasing the angle of incidence. Thus, the simulation results show that the artificial compound eyes with a wide FOV provide a superior focusing ability.

### Characterization of the Optical Performance of the Artificial Compound Eyes

**Fig. 6:**
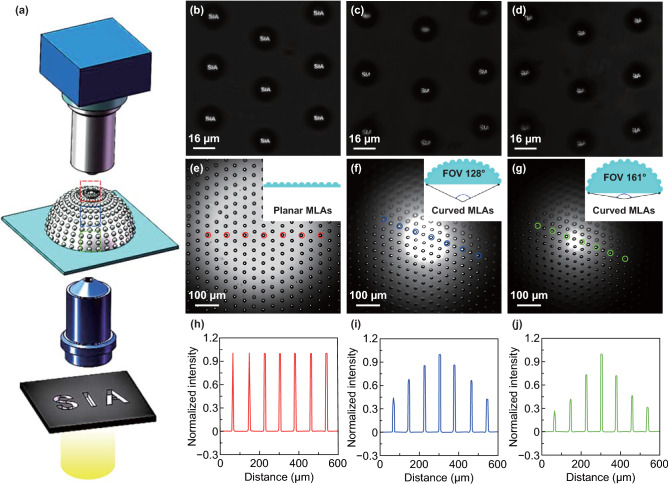
Characterization of the optical imaging performance and focusing ability of the artificial compound eyes. **a** Optical system for characterizing the artificial compound eyes. **b–d** Optical images of the letters “SIA” focused by MLAs distributed in the center, middle, and edge regions of the artificial compound eyes, as indicated at the areas enclosed by the red, blue, and green dashed lines in **a**. **e–g** Focusing ability test for the tunable artificial compound eyes with variable FOVs of 0°, 128°, and 161°, respectively. **h–j** Normalized light intensity distribution obtained from the focal spots indicated by the circled positions shown in (**e–g**)

The testing system for characterizing the optical imaging performance and focal ability of the artificial compound eyes is shown in Fig. 6a. The artificial compound eyes were positioned on a sample stage and moved along the optical axis. A black mask with transparent letters “SIA” was illuminated by a spot light source and zoomed via an objective lens (× 10). The projected image of the letters “SIA” was observed via a microscope equipped with a CCD camera. First, the optical imaging performance of the artificial compound eyes was investigated. The fabricated artificial compound eyes with a height of 0.68 mm, a diameter of 1.6 mm, a radius of curvature of 0.81 mm, and an FOV of 161° was used for the imaging test. The parameters of the H-MNLAs were introduced as follows: MLAs with an average diameter of 17.55 μm, height of 4.63 μm, focal length of 18.98 μm, and NA of 0.46; NLAs with an average diameter and height of 183.8 and 48.3 nm, respectively. Owing to the spherical distribution of the MLAs on the surface of eyeball, the focus positions of these lenses formed a spherical surface. As a result, the optical images were focused via the MLAs distributed at the center, middle, and edge regions of the artificial compound eyes, respectively. As shown in Fig. 6b–d, the images of the projected letters “SIA” produced by the array can be clearly observed through the CCD camera. This demonstrates the uniformity of the MLAs and the good optical imaging performance of the artificial compound eyes.

To investigate the focusing ability of the artificial compound eyes with the tunable FOV, the mask of the letters “SIA” was replaced by the mask of a light spot (Fig. S13). By the same focusing process, while varying the tunable deformation of the artificial compound eyes via the microfluidics chip, as shown in Fig. 6e–g, the focal points of lens at the center of the MLAs can be observed clearly as the MLAs film was deformed from a planar to a curved surface. The inset schematics illustrate the deformation state of the MLAs film for the artificial compound eyes, with the height, diameter, radius of curvature, and FOV values corresponding to Fig. 6f, g measuring 0.50 mm, 1.6 mm, 0.89 mm, and 128º, and 0.68 mm, 1.6 mm, 0.81 mm, and 161º, respectively. When the MLAs film was deformed from a planar surface to a curved surface, the microlenses distributed on the surface of the deformed eyeball function as a lens, and the light spot is amplified by the eyeball, with the result that the spot focused by the microlens becomes larger. Meanwhile, the phenomenon is aggravated by the divergence of the light source; the variation will be reduced if the parallel white light source is replaced by laser source, which has a better focusing property [39, 40]. The normalized light intensity distribution of the focal spots indicated by the circled positions in Fig. 6e–g is shown in Fig. 6h–j. The sharp intensity distribution indicates that the artificial compound eyes exhibit good uniformity of morphology and excellent optical focusing ability. In addition, it must be noted that the light intensities at the focal spots of the microlens decrease from the center region to the edge regions of the artificial compound eyes, with this phenomenon more evident in the artificial compound eyes with a wider FOV. This is because the incident angle is larger for the MLAs distributed in the edge regions than those in the center regions. Meanwhile, the angle was larger for the artificial compound eyes with a wider FOV, which results in the decrease in perpendicularity of incident light and light intensity of focal spots in the edge regions.

**Fig. 7:**
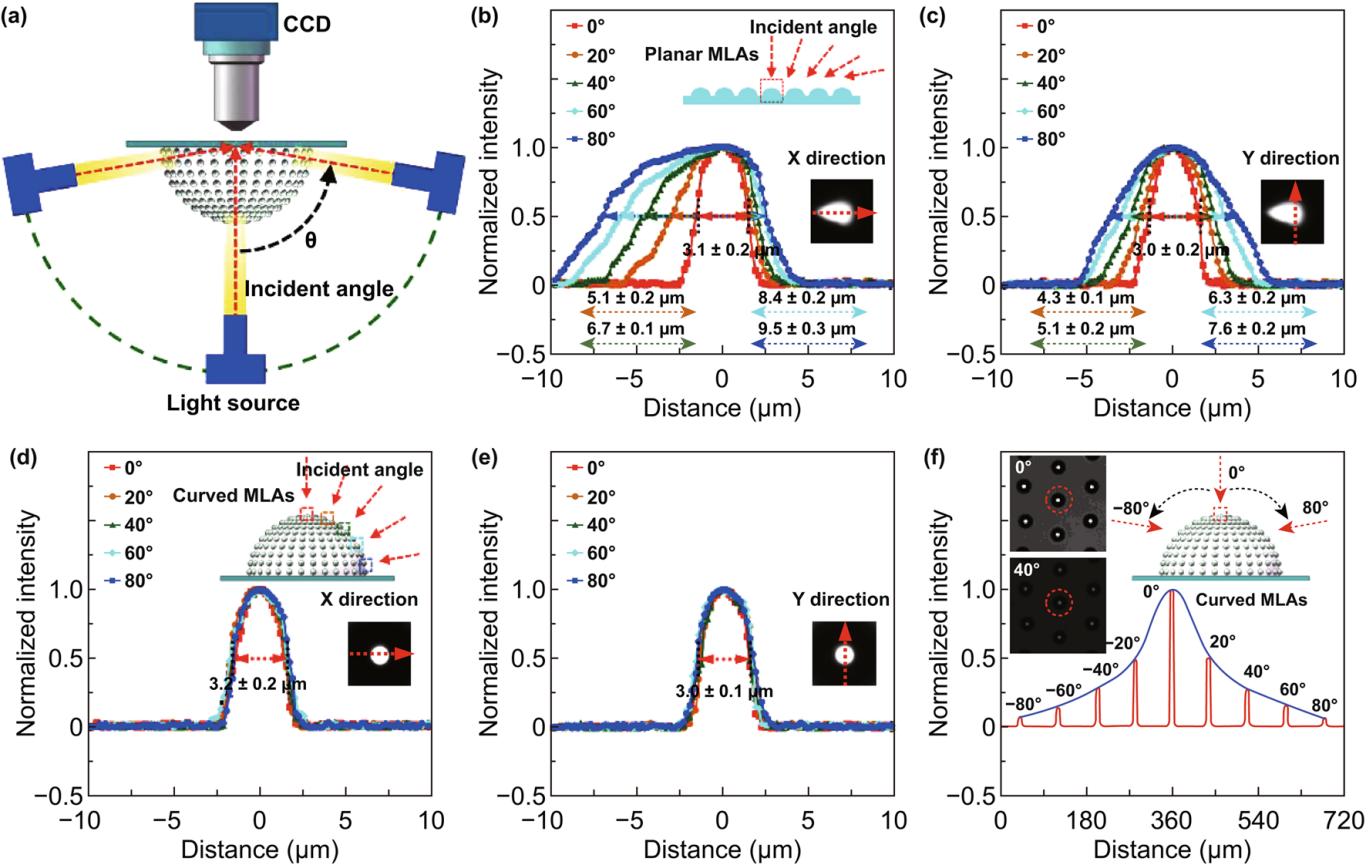
Characterization of the FOV properties of planar and curved MLAs of artificial compound eyes. **a** Schematic of the characterization of the FOV of the artificial compound eyes. **b** and **c** Normalized light intensity distribution (along x- and y-directions, respectively) of focal spots for a planar MLAs observed by the optical system at incident angles of 0°, 20°, 40°, 60°, and 80°. For each case, the inset optical image shows the focal spot for a single microlens of a planar MLAs at an incident angle of 40°. **d** and **e** Normalized light intensity distribution (along x- and y- directions, respectively) of focal spots for a curved MLAs observed at incident angles of 0°, 20°, 40°, 60°, and 80°. For each case, the inset optical image shows the focal spot for a single microlens of a curved MLA at an incident angle of 40°. **f** Normalized light intensity distribution of focal spot for the same single microlens of the curved MLAs observed at incident angles ranging from -80° to 80°. The inset optical images show the focal spot for the same MLAs at incident angles of 0° and 40°

The FOV is the most important optical parameter of the artificial compound eyes. Compared with planar MLAs, the dominant advantage of curved MLAs is the wider FOV, which will contribute to the artificial compound eyes having more various practical applications. As shown in Fig. 7a, an integrated optical system was fixed onto a 3D movable and rotatable sample stage to characterize the FOV properties of the planar and curved MLAs of artificial compound eyes. The focal spots of the MLAs were obtained by the optical system for different incident angles. Figure 7b, c shows the light intensity distribution of focal spots for a single microlens of planar MLAs observed at incident angles of 0°, 20°, 40°, 60°, and 80° along the *x*- and *y*-directions, respectively. As the incident angle increases, the distortion in the light intensity distribution along the symmetry axes becomes more evident. When the incident angle increases from 0° to 80°, the average full width at half-maximum (FWHM) intensity of the focal spots changes from 3.1 ± 0.2 to 9.5 ± 0.3 μm along the x-direction, and from 3.0 ± 0.2 to 7.6 ± 0.2 μm along the y-direction.

For comparison, planar MLAs were deformed to create curved MLAs for the FOV test of artificial compound eyes with a height and diameter of 0.77 mm and 1.6 mm, respectively. The theoretical FOV of the eyes was calculated as 176° based on Eq. 3. The light intensity distributions of the focal spots for curved MLAs at incident angles of 0°, 20°, 40°, 60°, and 80° along the *x*- and *y*-directions are shown in Fig. 7d and 7e, respectively. Almost no distortion occurred along the *x*- and *y*-directions as the incident angle was increased from 0º to 80º, with the average FWHM intensity at the focal spot measuring 3.2 ± 0.2 and 3.0 ± 0.1 μm along the *x*- and *y*-directions, respectively. The results indicate curved MLAs have a better focusing ability than planar MLAs under incident illumination at oblique angles. This result shows close agreement with the simulation results shown in Fig. 5g–j. Figure 7f shows the light intensity distribution of the focal spots for the same single microlens of the curved MLAs at incident angles ranging from − 80° to 80°. The light intensity for the same microlens decreased as the incident angle was increased. The result confirms that there are no evident imaging distortions caused by varying the incident angle, with the fabricated artificial compound eyes demonstrating a good and consistent imaging performance with an experimental FOV of 160° (− 80º to 80º), which has a negative deviation of only 9 % compared with the theoretical FOV (176º). This small deviation may be attributed to an assembling deviation occurring during the deformation process, which induces a deviation in the incident light.

## Conclusion

In conclusion, we proposed a novel method for the fabrication of waterproof artificial compound eyes with a variable FOV, which was realized based on the tunable deformation of a superhydrophobic MLAs film via a microfluidics chip. E-jet printing was used for fabricating the MLAs film, which consisted of a soft PDMS film covered with hierarchical MLAs and NLAs. The soft MLAs film was deformed from a planar surface to a curved surface through the precise control of liquid volume via a microfluidics chip. This resulted in artificial compound eyes with a wide tunable FOV, which ranged from 0° to 160°. Compared with other fabrication techniques, the proposed method provides a simple, flexible, and efficient approach for creating MLAs that offer potential advantages for numerous advanced and multipurpose microoptics applications. The fabricated MLAs can be used as planar MLAs or curved MLAs as required by various environments, including humid or even aqueous environments such as water. For example, the fabricated artificial compound eyes have the potential to be integrated with microoptical fibers to achieve a wide FOV for actual transmission imaging in medical endoscopy and microcavity environments. Furthermore, the hierarchical micro–nanostructures are not limited only to applications that require superhydrophobicity, and they can also be used in ultrasensitive flexible pressure sensors. In addition, the micro-/nanostructures can be used as a culture template for selective cell growth, which is applicable for cell patterning, tissue engineering and drugs screening. In the future, the techniques presented herein for fabricating hierarchical micro–nanostructures are worthy of further research due to their widespread potential for micro–nanofabrications and devices.

## Electronic Supplementary Material

Below is the link to the electronic supplementary material.

Supplementary material 1 (MP4 1039 kb)

Supplementary material 2 (MP4 427 kb)

Supplementary material 3 (MP4 333 kb)

Supplementary material 4 (PDF 793 kb)
