# Supplementary material for: Fabrication of Waterproof Artificial Compound Eyes with Variable Field of View Based on the Bioinspiration from Natural Hierarchical Micro–Nanostructures
Source: Nanomicro Lett. 2020 Aug 15;12:166. doi: 10.1007/s40820-020-00499-x (PMC7770831; doi:10.1007/s40820-020-00499-x)
Supplement: Supplementary file 4 — Supplementary material 4 (PDF 793 kb) [file 40820_2020_499_MOESM4_ESM.pdf]

Supporting Information for

Waterproof Artificial Compound Eyes with Variable Field of View based on the Bioinspiration from Natural Hierarchical Micro-Nano Structures

Peilin Zhou[1,2,3], Haibo Yu[1,2,*], Ya Zhong[1,2,3], Wuhao Zou[1,2,3], Zhidong Wang[1,4], Lianqing Liu[1,2,*]

[1]State Key Laboratory of Robotics, Shenyang Institute of Automation, Chinese Academy of Sciences, Shenyang 110016, People's Republic of China

[2]Institutes for Robotics and Intelligent Manufacturing, Chinese Academy of Sciences, Shenyang 110169, People's Republic of China

[3]University of Chinese Academy of Sciences, Beijing 100049, People's Republic of China

[4]Department of Advanced Robotics, Chiba Institute of Technology, Chiba 275-0016, Japan

*Corresponding authors. E-mail: yuhaibo@sia.cn (Haibo Yu), lqliu@sia.cn (Lianqing Liu)

Supplementary Figures

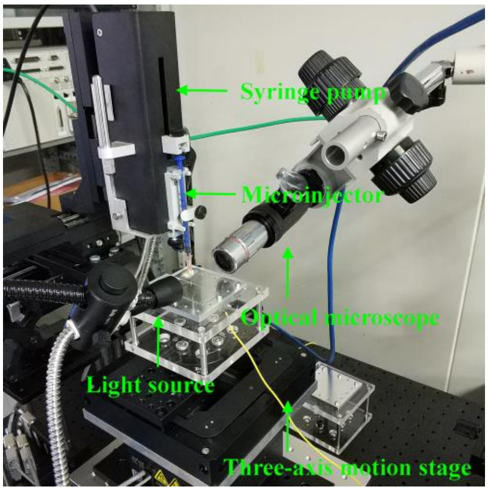

Fig. S1 Optical image of the homebuilt measurement system for characterizing the water contact angles (WCAs) of the samples

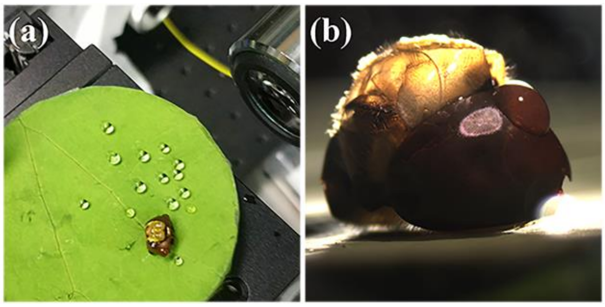

Fig. S2 Optical images of the measurement of WCAs on the surface of natural dragonfly eyes

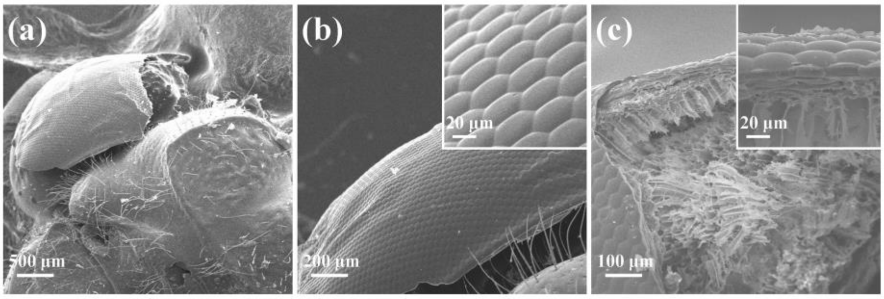

Fig. S3 SEM images of compound eyes of natural dragonfly

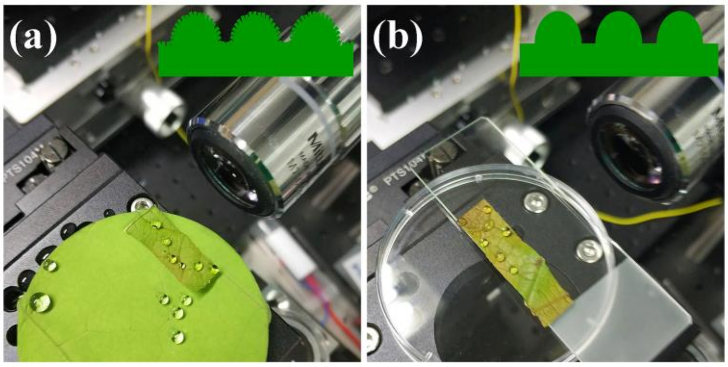

Fig. S4 Optical images of the measurement of WCAs on the surface of natural lotus leaf. (**a**) Lotus leaf was treated via conventional drying process at the room temperature (20 °C). (**b**) Lotus leaf was treated via heat treatment at 120 °C for 30 min after the conventional drying process

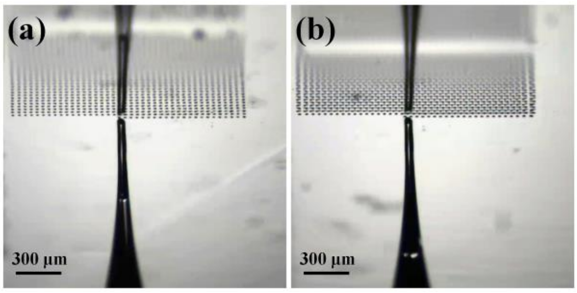

Fig. S5 Optical images in tilted view angle of the printing process for the fabrication of MLAs on PDMS film

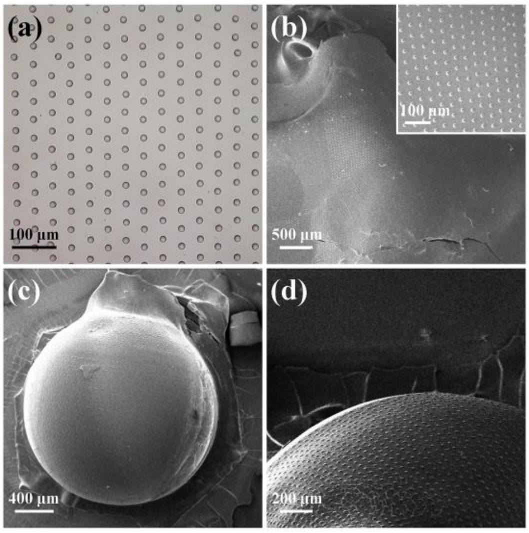

Fig. S6 Optical image and SEM images of MLAs film for the fabrication of artificial compound eyes. (a) The optical microscope image of MLAs fabricated on PDMS film. (b) The SEM image of the bending state of the soft MLAs film. (c) and (d) SEM images of the fabricated artificial compound eyes.

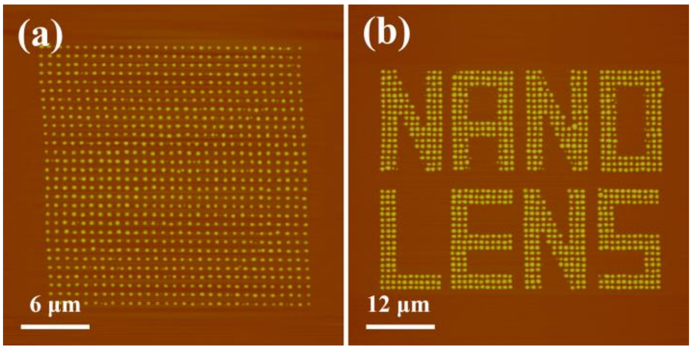

Fig. S7 AFM images of NLAs fabricated via the DOD printing under the stable cone-jet mode [S1]. (**a**) NLAs contain nanolenses of 900 (30 × 30), the average diameter and height of which were 384 nm, and 73 nm, respectively. (**b**) NLAs of complex shapes "NANO LENS" consist of 1016 nanolenses with homogeneous sizes, the average diameter and height of which were 840 and 153nm

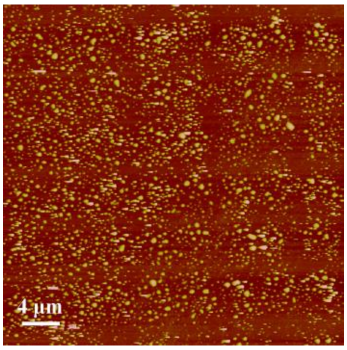

Fig. S8 AFM image of NLAs with more than $1×10^4$ nanolenses fabricated via the CR printing under the electrospray mode in ~0.2 s

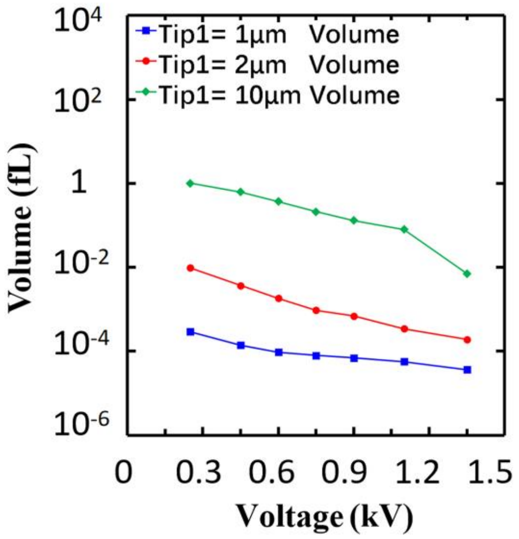

Fig. S9 Statistical analysis of the volume of the nanolenses printed by nozzles with diameters of 1, 2, and 10 μm under different applied voltages

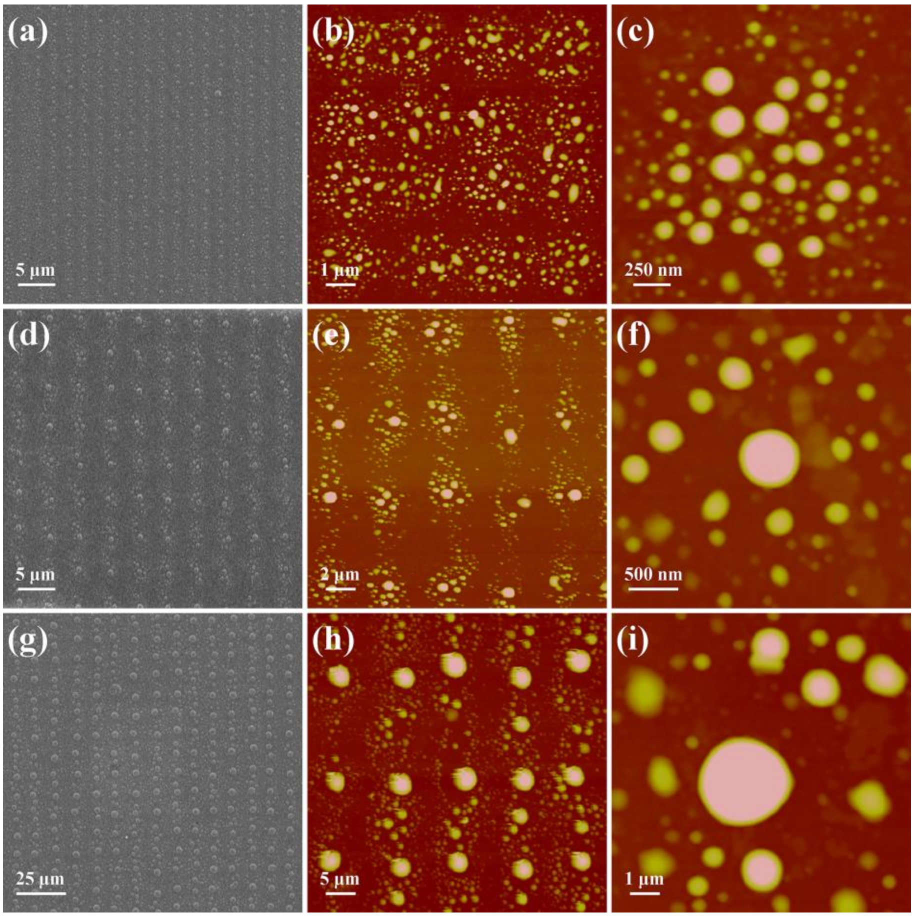

Fig. S10 SEM and AFM images of the nanolenses printed via different printing nozzles under the electrospray printing mode, respectively. (**a–c**) printing nozzles with diameters of 1 µm, (**d–f**) 2µm, (**g–i**) 10µm

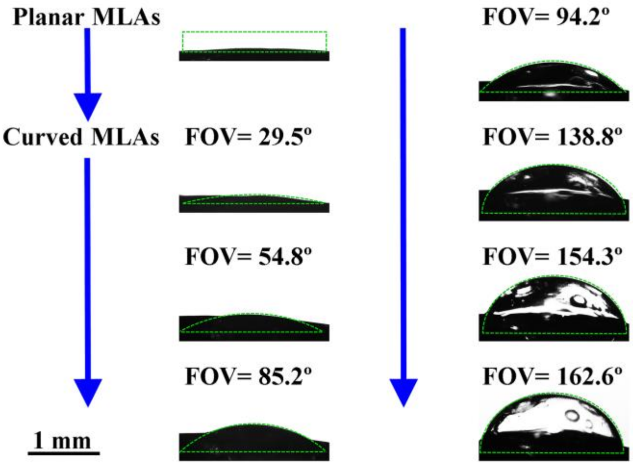

Fig. S11 The optical images in cross-sectional view angle of eight different configurations when the soft MLAs film was deformed from a planar surface to a curved surface for the tunable deformation of artificial compound eyes.

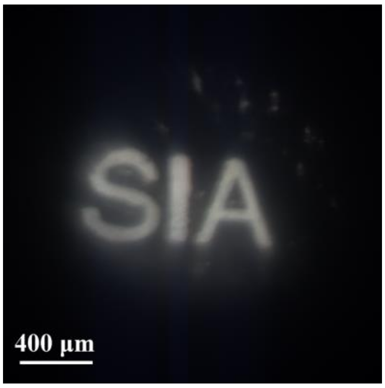

Fig. S12 The optical image of the letters “SIA” focused by the eyeball of the artificial compound eyes.

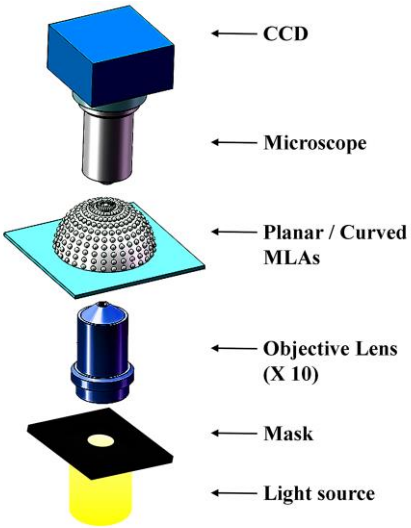

Fig. S13 Schematic of the optical system for characterization of focusing property of the artificial compound eyes

Supplementary Reference

[S1] P. Zhou, H. Yu, W. Zou, Y. Zhong, X. Wang, Z. Wang, L. Liu, Cross-scale additive direct-writing fabrication of micro/nano lens arrays by electrohydrodynamic jet printing. Opt. Express **28**(5), 6336-6349 (2020). https://doi.org/10.1364/oe.383863
